# Central Venous Line Insertion Revealing Partial Anomalous Pulmonary Venous Return: Diagnosis and Management

**DOI:** 10.1155/2017/3218063

**Published:** 2017-05-29

**Authors:** Bashar Alzghoul, Ayoub Innabi, Aditya Chada, Ahmad R. Tarawneh, Krishna Kakkera, Khaled Khasawneh

**Affiliations:** ^1^Department of Internal Medicine, University of Arkansas for Medical Sciences, Little Rock, AR, USA; ^2^Division of Pulmonary and Critical Care Medicine, University of Arkansas for Medical Sciences, Little Rock, AR, USA

## Abstract

Central venous line malposition is a well-known complication of line insertion. Rarely, it can be mal-positioned in an anomalous pulmonary vein. We present an unusual case of a 56-year-old woman that was found to have partial anomalous pulmonary venous return on central venous line insertion. In this report, we describe a systematic approach to diagnosis and management of this unusual situation.

## 1. Introduction

Partial anomalous pulmonary venous return (PAPVR) is a rare congenital anomaly that occurs during embryologic development when at least one pulmonary vein fails to communicate with the left atrium; instead, it drains directly or indirectly in the right atrium. It remains asymptomatic most of the time and is discovered incidentally or postmortem [1]. There have been few reports in literature of PAPVR discovered after central venous line (CVL) insertion. We report a 56-year-old woman that was found to have left upper PAPVR on CVL insertion. In addition, we conducted literature review of similar cases reported before and suggested a systematic approach to diagnosis and management.

## 2. Case Presentation

**Figure 1:**
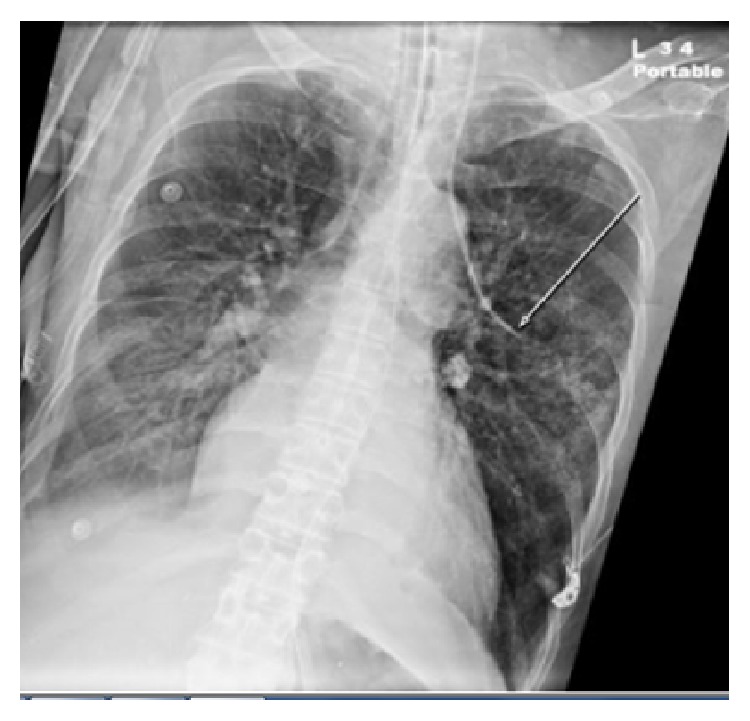
Single frontal view CXR showing the tip of the left IJ CVL extending left of the aortic arch towards the left lung field.

**Figure 2:**
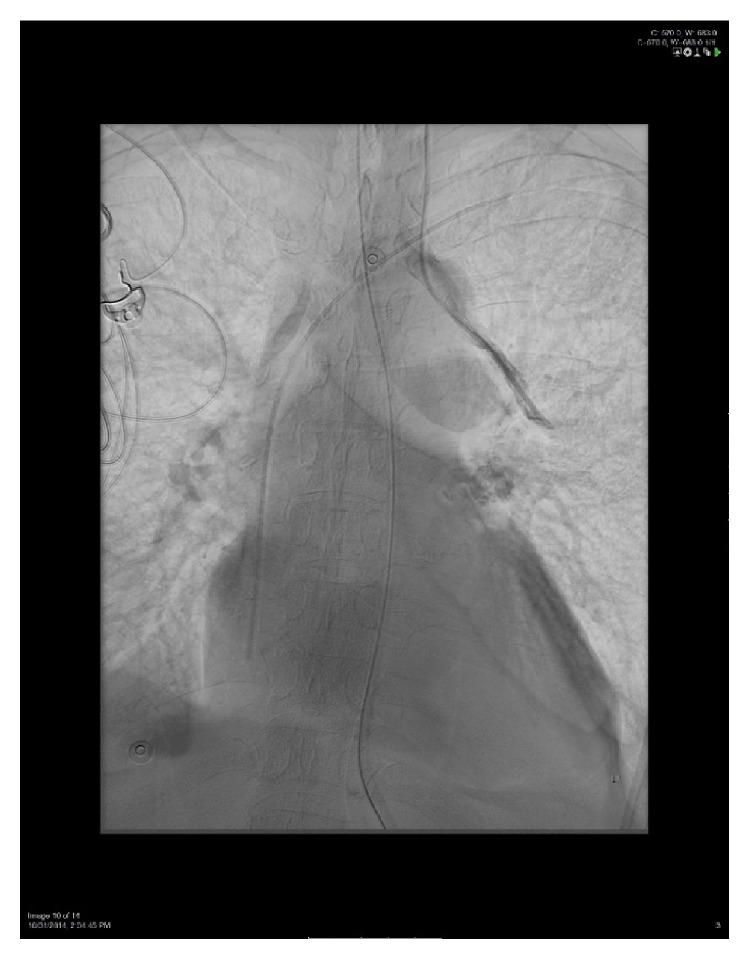
Fluoroscopy study showing left IJ CVL extending through the anomalous pulmonary vein.

A 56-year-old woman with a medical history of multiple sclerosis, quadriplegia, sacral decubitus ulcer, chronic osteomyelitis, chronic deep vein thrombosis (DVT), chronic steroids use, and chronic Foley catheter use presented to the emergency department with severe sepsis due to multiple potential sources including urinary tract infection, soft tissue infection, or osteomyelitis. Vital signs were significant for blood pressure of 85/59 mmHg, heart rate of 63 beats per minute, respiratory rate of 33 breaths per minute, and oxygen saturation of 83% on room air. At the beginning, patient declined intubation so she was placed on 100% oxygen via nonrebreather mask. Arterial blood gas (ABG) on 100% oxygen was 7.29/49/55. Initial chest X-ray (CXR) showed small bilateral pleural effusions with suspected left lower lobe infiltrate. Despite intravenous (IV) fluid resuscitation, broad spectrum IV antibiotics, and stress dose steroids, patient became more hypotensive and IV norepinephrine was started through a right femoral CVL that was placed in the emergency department. Patient was started on heparin drip for suspected pulmonary embolism that was stopped later due to large bleeding from femoral line site and consideration of pneumonia as the cause of hypoxemia. Patient agreed later to be intubated and decision was made to remove femoral line and place a 7.0 Fr triple-lumen CVL through the left internal jugular vein (IJ) over a guidewire and under full ultrasound (U/S) guidance, which was placed easily with no complications. A CXR was obtained to confirm placement which showed that the catheter tip did not cross the midline; instead, it was extending towards the left of the aortic arch probably into an anomalous vein as read by the radiologists (Figure 1). Ultrasound examination of the neck showed proper catheter placement in the left IJ. A pressure transducer was connected which showed a venous wave form with a pressure of 10 mmHg. ABG obtained from the catheter on continuous mechanical ventilation (FiO_2_ = 50%, positive end expiratory pressure = 5 CM H_2_O) showed PaO_2_ of 102 mmHg compared to 79 mmHg from radial arterial site. A transthoracic echocardiography, which was done to evaluate cardiac function, did not show evidence of atrial septal defect or any other cardiac malformation. Fluoroscopy study was used to place a left peripherally inserted central catheter (PICC) and to confirm the location of the left IJ CVL which demonstrated contrast injection in an anomalous pulmonary vein (Figure 2). We concluded that the patient has partial anomalous pulmonary venous return (PAPVR) and the left IJ CVL was removed. The PICC line was used instead for central access.

## 3. Discussion

Central venous lines (CVLs) insertion is a very common procedure that is performed by practitioners of different specialties for varied indications. Among these indications are drug administration, parenteral nutrition, renal replacement therapy, cardiac catheterization, and trans-venous pacing of the heart [2]. Malposition of CVL insertion is relatively common due to operator and technical factors in part. However, it can be due to congenital and acquired abnormalities of the venous system that leads to abnormal positioning of the line. In some cases, CVL malposition can lead to catastrophic complications [3, 4]. Thus, it is critically important for practitioners to identify malposition of CVLs and take evidence based appropriate actions depending on the situation.

CXR is routinely used to confirm the location of the CVL, which ideally should be at the junction between the superior vena cava (SVC) and the right atrium. There are multiple radiographic landmarks that can be utilized on CXR to confirm that the CVL is in the right location [5]. In our case, we encountered an unusual location of the CVL on the CXR where the catheter tip did not cross the midline; instead, it was extending laterally towards the left lung field, left of the aortic arch. The CVL was clearly mal-positioned, but the question that we had was where exactly was the line inserted: Was it inserted in the carotid artery? Does the patient have a persistent left SVC?

We decided to follow a systematic approach that showed the CVL was inserted in an anomalous pulmonary vein. This finding might be encountered by other practitioners in critical situations where someone needs to make fast decisions, so it is important to be familiar with the anatomy.

**Figure 3:**
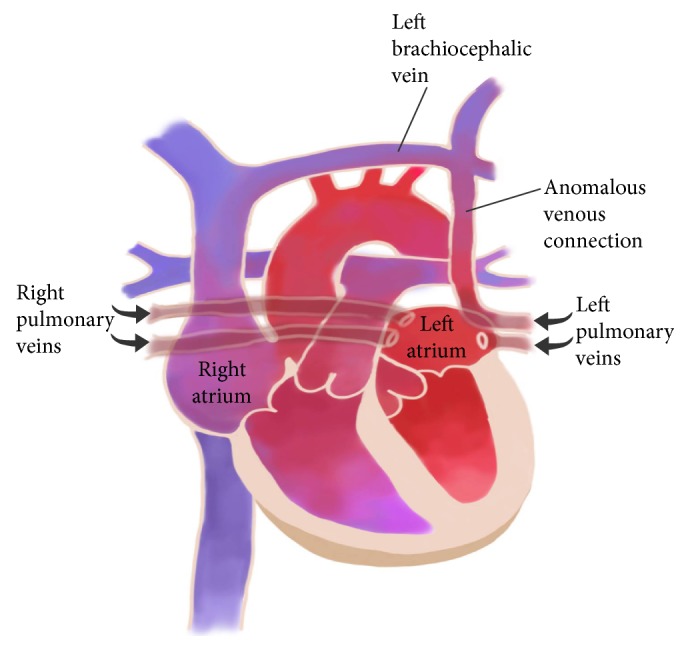
Schematic illustration of the anatomy of the CVL inserted accidentally in an anomalous pulmonary vein connected to the right atrium via the brachiocephalic vein.

The pulmonary veins originally arise from a common pulmonary vein that buds from the left atrium during the embryologic development. It then joins the splanchnic venous plexus in the lung buds. If any or all of the pulmonary veins fail to connect to the left atrium, it will eventually divert the blood directly or indirectly to the right atrium [1, 6].  Figure 3 shows a schematic view of left upper partial anomalous pulmonary venous return (PAPVR). On cadaveric studies, PAPVR was reported in up to 0.7% of the cases [1]. It was first described by Winslow in 1739 [7, 8]. There are scarce cases in literature of accidental diagnosis of PAPVR in adults after CVL insertion. We did a literature review using PubMed and Google scholar search engines using the key words (Central Venous) and (partial anomalous). We included cases of adult patients who were diagnosed to have PAPVR after CVL insertion and ended up with 10 cases. As illustrated in Table 1, we managed to summarize the approach that was followed in these cases. We tried in this article to highlight the diagnostic and management approach followed in other reported cases and suggest a systematic approach to follow when encountering similar situation.

First we used the U/S to examine the course of the CVL in the neck to confirm whether the CVL was inserted in the IJ vein or in the carotid artery. Of note, U/S examination of the chest can be used sometimes to localize the tip of CVL; however, it needs an experienced operator and may not detect the final intrathoracic placement of the CVL all the time [9]. Second, we connected the CVL to a pressure transducer which showed a venous wave form. Among the cases reported in literature, Wylam and Schmidt [6] were the only to describe pulsating arterial wave form. They attributed that to wedging of the tip of the catheter in a pulmonary venous branch, which would subsequently reflect the pressure tracing from that of the upstream pulmonary artery. However, in their case, the patient had moderate pulmonary hypertension diagnosed by heart catheterization. Third, we obtained ABG from the CVL and from arterial site. In our case, the PaO2 was paradoxically higher from the CVL compared to arterial sample. This paradoxical finding was reported in four of the 10 cases [6, 10–12]. In addition, PO_2_ was reported to be high in four other cases, but no arterial sample was obtained to compare [13–16]. The high PO_2_ can be explained by the fact that the catheter tip is collecting blood directly from the pulmonary venous blood flow before intermixing with systemic venous blood [15]. The fourth step would be to confirm placement via definitive radiologic method. In our case, since we decided to substitute the CVL with a PICC, we used fluoroscopy studies to define the anatomy. From this, it might be unnecessary to use CT/CTA only to establish the diagnosis of CVL insertion in an aberrant pulmonary vein unless it was indicated for some other reason. Instead, one can use the constellation of findings described above and confirm the diagnosis using simple fluoroscopy studies.

There are no enough cases in literature to examine the safety of using the CVL inserted in an anomalous pulmonary vein. In our case, we opted to remove the catheter since we had alternative options. Other authors opted for reposition of the catheter under fluoroscopy guidance [6, 14, 17]. However, Khanna and colleagues [11] reported using the CVL safely for two days as venous access. In addition, Grillot and colleagues [12] reported using it for seven days for hemodialysis.

## 4. Conclusion

It is important to be familiar with PAPVR as a cause of CVL malposition. When encountered, the systematic approach can help establish the diagnosis. This includes ultrasound confirmation of venous access, checking blood gases from the CVL and concurrently from an arterial site which is expected to show high PO_2_ that might be paradoxically higher than the arterial side, checking the pressure wave, and using fluoroscopy to confirm radiologically.

## Figures and Tables

**Table 1:** Summary of the approach followed in 10 cases reported in literature of adult patients found to have PAPVR on CVL insertion.

Article	Year	Side	Indication	Pulmonary systemic connection site	Paradoxical paO_2_	Wave form	Fluoroscopy	CTA/CT chest	U/S confirmation	Pulmonary HTN	Management
Wylam and Schmidt [6]	1990	Left IJ	Sepsis	Left upper pulmonary vein to left brachiocephalic	√	Pulsatile	√	No	n/a	√	Repositioned

Cheng et al. [10]	2002	Left IJ	Sepsis	Left upper pulmonary vein to left brachiocephalic	√	Venous	√	No	√	n/a	Removed

Townley [13]	2003	Left IJ	Venous access	Left lung	High PO_2_ but no arterial sample to compare	Venous	No	No	n/a	n/a	Removed

Chintu et al. [18]	2008	Left IJ	Hemodialysis	Left upper pulmonary vein to left brachiocephalic	n/a	n/a	√	No	n/a	n/a	Removed

Javangula et al. [14]	2010	Left IJ	Sepsis	Left upper pulmonary vein to left brachiocephalic	High PO_2_ but no arterial sample to compare	Venous	√	CTA	√	No	Repositioned

Habito and Kalva [17]	2011	Left IJ	Chemotherapy	Right upper pulmonary vein to SVC	n/a	n/a	√	CTA	n/a	n/a	Repositioned

Brandt and Artmeier-Brandt [15]	2015	Left subclavian	Venous access in the setting of trauma	Left upper pulmonary vein to left brachiocephalic vein	High PO_2_ but no arterial sample to compare	Venous	No	CTA	n/a	n/a	n/a

Khanna et al. [11]	2014	Left IJ	Intraoperative venous access	Left upper pulmonary vein to left brachiocephalic	√	Venous	No	Regular CT without contrast	n/a	n/a	Used for 2 days

Chirinos et al. [16]	2014	Left IJ	Hemodialysis	Left upper pulmonary vein to left brachiocephalic	High PO_2_ but no arterial sample to compare	Venous	Yes	CTA	n/a	n/a	Removed

Grillot et al. [12]	2016	Left IJ	Hemodialysis	Left upper pulmonary vein to left brachiocephalic	√	Venous	No	CTA	√	n/a	Used for 7 days

n/a: not available.
